# What is cumulative cultural evolution?

**DOI:** 10.1098/rspb.2018.0712

**Published:** 2018-06-13

**Authors:** Alex Mesoudi, Alex Thornton

**Affiliations:** Human Behaviour and Cultural Evolution Group, College of Life and Environmental Sciences, University of Exeter, Exeter, UK

**Keywords:** animal culture, cultural evolution, cumulative culture, innovation, social learning

## Abstract

In recent years, the phenomenon of cumulative cultural evolution (CCE) has become the focus of major research interest in biology, psychology and anthropology. Some researchers argue that CCE is unique to humans and underlies our extraordinary evolutionary success as a species. Others claim to have found CCE in non-human species. Yet others remain sceptical that CCE is even important for explaining human behavioural diversity and complexity. These debates are hampered by multiple and often ambiguous definitions of CCE. Here, we review how researchers define, use and test CCE. We identify a core set of criteria for CCE which are both necessary and sufficient, and may be found in non-human species. We also identify a set of extended criteria that are observed in human CCE but not, to date, in other species. Different socio-cognitive mechanisms may underlie these different criteria. We reinterpret previous theoretical models and observational and experimental studies of both human and non-human species in light of these more fine-grained criteria. Finally, we discuss key issues surrounding information, fitness and cognition. We recommend that researchers are more explicit about what components of CCE they are testing and claiming to demonstrate.

## 1. Introduction

Anthropologists, biologists and psychologists have long been engaged in a search to discover the traits that make us uniquely human. Why have we, alone in the animal kingdom, created art and literature, socio-political systems that permit large-scale cooperation, and the scientific and technological knowledge to colonize the whole planet and explore space? Over the years, many candidates, including tool-making, episodic memory and semantic communication, have fallen by the wayside as researchers have uncovered hitherto unknown abilities in other animals [1].

Today, a leading front-runner for the key to human success is cumulative culture, or cumulative cultural evolution (CCE). This concept was brought to prominence in the 1990s by Boyd & Richerson [2] and Tomasello [3] to contrast human culture with the culture of non-human species. Even then there was evidence for both social learning and cultural traditions in non-human species, and this evidence has amassed in the years since. Many species across multiple taxa learn from one another, and in such a way that can generate behavioural differences between groups of individuals [4–9]. However, Tomasello argued that only humans could ‘accumulate modifications over time’ where

some individual or group of individuals first invented a primitive version of [an] artifact or practice, and then some later user or users made a modification, an ‘improvement,’ that others then adopted perhaps without change for many generations, at which point some other individual or group of individuals made another modification, which was then learned and used by others, and so on over historical time in what has sometimes been dubbed ‘the ratchet effect’ [3, p. 5].

A ratchet is a device with angled teeth that allows a bar or cog to move in one direction only. Here, it is a metaphor for the accumulation of increasingly effective modifications without reverting back to prior, less effective states. Boyd & Richerson highlight the consequences of CCE:

In contrast [to non-human species' cultural traditions], human cultures do accumulate changes over many generations, resulting in culturally transmitted behaviors that *no single human individual could invent on their own.* Even in the simplest hunting and gathering societies people depend on such complex, evolved knowledge and technology. To live in the arid Kalahari, the !Kung San need to know what plants are edible, how to find them during different seasons, how to find water, how to track and find game, how to make bows and arrow poison, and many other skills. The fact that the !Kung can acquire the knowledge, tools, and skills necessary to survive the rigors of the Kalahari is not so surprising—many other species can do the same. What is amazing is that the same brain that allows the !Kung to survive in the Kalahari, also permits the Inuit to acquire the very different knowledge, tools, and skills necessary to live on the tundra and ice north of the Arctic circle, and the Ache the knowledge, tools, and skills necessary to live in the tropical forests of Paraguay. There is no other animal that occupies a comparable range of habitats or utilizes a comparable range of subsistence techniques and social structures. [2, p. 80]

The italicized phrase in this quotation highlights a commonly cited consequence or criterion for CCE that its products exceed what a single individual could invent alone. The rest of the quotation represents a typical argument for the adaptiveness of CCE.

Over 20 years later, CCE remains a frequently cited rubicon between human and non-human cognition and culture [10–13], but has not gone unchallenged. There have been claims of CCE in chimpanzees [14], baboons [15], macaques [16], New Caledonian crows [17], pigeons [18] and in the songs of some birds [19] and cetaceans [20]. In some cases [e.g. 18], almost identical experimental designs are used to demonstrate CCE in a non-human species as those used to demonstrate CCE in humans [21].

Leaving non-human species aside, there is also debate within the human evolutionary sciences over the importance, or even existence, of CCE. Proponents of ‘cultural attraction’ (e.g. [22]) have argued that CCE is less important for explaining human cultural diversity and change than claimed by other cultural evolution researchers (e.g. [23]) and focus more on intuitively attractive cultural traditions that *do not* exceed what one individual could invent or reconstruct alone. Some evolutionary psychologists, meanwhile, deny any meaningful role for culture in generating human behavioural diversity, instead focusing on how behaviour is generated by genetic programmes that evoke different behaviour in different environments [24,25]. According to this view, complex behaviour arises from cumulative *genetic* evolution plus sophisticated genetically evolved individual cognition [26], not CCE.

These twin debates—one over whether CCE is found in non-human species and the other over the importance of CCE in explaining human ecological success—are hampered by the multiple ways in which CCE is used and defined. Some of these definitions are listed in electronic supplementary material, table S1. While there are many commonalities, there is also much variation. Often, these different senses of CCE are not made explicit, leading to confusion or seemingly contradictory claims.

Our aim is to review how researchers typically use, test and define CCE to resolve, or at least highlight, this ambiguity. We first suggest a set of core criteria that are necessary for a population to exhibit CCE. We then specify a set of extended criteria which may or may not be present. We reinterpret previous theoretical models and empirical findings in light of these criteria, suggest that different cognitive mechanisms may underlie different forms of CCE and highlight problematic concepts. We recommend that researchers are clearer about which criteria they are studying and avoid treating CCE as a unitary rubicon separating human and non-human species.

## 2. Core criteria

Our core criteria follow the definition of CCE provided in Tomasello's quotation above. We suggest that the minimum requirements for a population to exhibit CCE are (i) a change in behaviour (or product of behaviour, such as an artefact), typically due to asocial learning, followed by (ii) the transfer via social learning of that novel or modified behaviour to other individuals or groups, where (iii) the learned behaviour causes an improvement in performance, which is a proxy of genetic and/or cultural fitness, with (iv) the previous three steps repeated in a manner that generates sequential improvement over time.

The first criterion provides a source of behavioural variation in the form of either the emergence of entirely new behaviour or modification of existing behaviour. This could occur via asocial learning (e.g. associative learning or higher-level problem-solving or creativity) or collective learning, where behavioural novelty arises from the interactions between individuals in groups [27,28]. Variation may also be introduced by random copying error or other stochastic processes. Without the introduction of behavioural variation, there can be no change over time, only stasis, which would clearly not constitute CCE.

The second criterion specifies that the behavioural variant must be passed to others via social learning. If this did not occur, then the innovation would be lost when the innovating individual died or the innovating group disbanded. This again would not count as CCE (nor as culture more generally, thus justifying the word ‘cultural’ in the term CCE).

The third criterion specifies that the learned behavioural variant must enhance some measure of performance, which is a proxy for inclusive genetic and/or cultural fitness. This is implied in Tomasello's use of the term ‘improvement’ and Boyd & Richerson's description linking CCE to ecological adaptation. Several definitions in electronic supplementary material, table S1 also mention ‘improvement’, although this is seldom itself explicitly defined. By ‘performance’, we mean the characteristics of the socially learned trait that are maximized or desired according to the neurobiological, cognitive, emotional and other evaluative mechanisms of individuals. Examples of performance measures might include the efficiency of migratory routes or extractive foraging, the durability and sharpness of cutting tools, or the aesthetic attractiveness of art or dress styles. In some cases of CCE, especially in non-human species, this increase in performance will increase genetic fitness in terms of direct or indirect reproductive success (i.e. inclusive fitness). In other cases, especially in post-demographic transition human societies, it is harder to see genetic fitness benefits of CCE. Here, we may speak instead of ‘cultural fitness’ where CCE may not maximize—and may even be detrimental to—genetic fitness [29,30]. The notion of fitness/improvement is complex and is revisited below.

The final criterion states that innovation and social learning must be repeated over time to generate sequential improvement in performance. Although admittedly ambiguous, the terms ‘repeated’ and ‘sequential’ are intended to rule out cases where a single behavioural variant spreads within a population, perhaps to fixation, with no further modification or improvement. For instance, meerkat pups learn from adults to eat hard-boiled eggs, a novel experimentally introduced food source, but there is no further modification to this tradition [31]. As Tomasello notes, there may be a period of stasis before further modification or improvement, which can only be detected with long-term historical data. This final core criterion justifies the word ‘cumulative’ in the term CCE.

We can contrast our core CCE criteria with the following cases of non-CCE which do not fulfil the criteria: (a) asocial or collective learning with no social learning beyond the immediate individual or group, which would produce improvement in performance that is lost when individuals die or groups disband, (b) improvement via genetic adaptation by natural selection, where the increase in fitness is via beneficial genetic mutations and the transmission is genetic and (c) cultural evolution that is non-cumulative, where fitness-neutral learned behaviours are transmitted via social learning. The latter may include changes in traits such as first names in humans [32], or changes in birdsong [33], both of which fit theoretical expectations of neutral drift.

Finally, note that our core criteria justify the word ‘evolution’ in the term CCE, by providing a system of descent with modification that bears parallels with genetic evolution [34]. Criterion (i) provides variation, criteria (ii) and (iv) provide an inheritance system, while criterion (iii) provides a means of adaptation to local environments, sometimes called ‘cultural adaptation’ [35]. This is not to imply that CCE is identical to cumulative genetic evolution [36]: for example, the generation of fitness-enhancing innovations by asocial learning may be very different to blind genetic mutation.

## 3. Extended criteria

While all definitions of CCE in the literature listed in electronic supplementary material, table S1 explicitly or implicitly include our core criteria, some include additional criteria that we view as extensions of the core criteria.

### (a) Multiple functionally dependent cultural traits

Our core criteria could apply to a single behavioural trait that is refined over time to generate repeated improvement in the same performance measure. For example, a navigation route towards the same fixed point might be increasingly refined to become more efficient over time [18]. An extension of this would be where multiple socially learned behavioural traits are chained together to generate repeated improvement in the same performance measure, with each step functionally or sequentially dependent on the previous steps [37]. As an example of functional dependence, Enquist *et al*. [38] suggest the Four Colour Conjecture in mathematics, for which there were a series of successive partial solutions each building and improving on the previous one. This functional dependence of multiple cultural traits is mentioned in some definitions in electronic supplementary material, table S1 (e.g. ‘To make the process of cultural accumulation realistic, we specified that innovations were contingent upon earlier discoveries' [39] or ‘Dependencies refer to relationships between elements, such that the presence of one cultural element affects the likelihood that another element appears or disappears’ [38]).

### (b) Diversification into multiple lineages

Our core criteria focus on a single lineage of an increasingly refined behavioural trait. Our first extended criterion, functional dependence, can also occur with a single lineage of linked traits. A further extension would be diversification, with parallel lineages arising when one lineage branches into multiple lineages. These lineages may, at least initially, be alternative means of maximizing the same performance measure, such as the bow-and-arrow and the spear-thrower as alternative means of projectile hunting [40]. Diversification is mentioned in some definitions in electronic supplementary material, table S1 (e.g. ‘[An] important characteristic of cumulative technological evolution [is] diversification of tool design’ [17] or ‘[a] fundamental propert[y] of human cumulative culture [is] lineage specificity, with different kinds of structure emerging in different chains') [15]. Diversification may occur within individuals (a single individual has knowledge of both bows and spear-throwers), between individuals within the same group (some individuals use bows, others spear-throwers) or between semi-isolated groups within a larger population, paralleling mechanisms of speciation in genetic evolution (e.g. sympatric and allopatric speciation).

### (c) Recombination across lineages

Once there are multiple cultural lineages, we may see recombination of traits across those lineages. Some definitions of CCE in electronic supplementary material, table S1 refer to this recombination (e.g. ‘The paradigmatic case of ratcheting is when an individual adds an existing technique used in a different context … to an existing technique, and integrates them functionally’ [41]).

In human CCE, recombination can be explicitly measured in the patent record, where patent filers must cite any previous patents (prior art) upon which their patent is based. Youn *et al*. [42] found that the proportion of all patents that constitute recombination, defined as the combination of two or more existing patents, has increased since 1870, becoming much more common than non-recombination inventions, which represent entirely new technology classes or cite only a single previous patent.

### (d) Cultural exaptation

The previous three extended criteria could occur with the same performance measure or function. The bow-and-arrow and spear-thrower, for example, are divergent lineages of functionally dependent traits that both fulfil the function of launching projectiles. In other cases, the previous three criteria could result in a *change* of function: a trait that originally culturally evolved to maximize one performance measure may be used to fulfil another. This resembles exaptation in genetic evolution [43], and we label this cultural exaptation. There are numerous examples from human CCE of technologies originally designed for one function eventually becoming more widely used to fulfil another [44,45], such as viagra, originally invented as a treatment for angina [46], or the use in ship rudders of iron hinges originally used for cathedral or castle doors [47].

### (e) Cultural niche construction

We have so far assumed that performance measures and fitness proxies are independent of the CCE process and its products, with CCE resulting in increasing cultural fitness (e.g. sharper blades). While cultural exaptation involves a *change* in function, it is also possible that CCE can itself modify and create fitness proxies. This would open up entirely new design spaces that cannot be reached without prior CCE. This can be seen as a form of cultural niche construction [48], where CCE modifies and creates its own selection pressures. For example, the invention of automobiles in the early twentieth century opened up a new design space for rubber tyres, which tyre manufacturers rapidly explored [49].

## 4. Models of cumulative cultural evolution

Electronic supplementary material, table S2 lists models that have attempted to capture the dynamics of CCE. The most influential model, Henrich's [50] ‘Tasmanian’ model, features all of our core criteria and none of our extended criteria. There is a single fitness proxy, *z*, and a single behavioural trait that can be increasingly refined to reach higher values of *z*. Each individual of each new generation attempts to copy the trait of the individual in the previous generation with the highest *z*. Improvement occurs via ‘lucky guesses or errors': occasionally an individual generates a behaviour with a higher *z* than the best demonstrator. Henrich used this model to highlight the limits that population size places on CCE, as populations that are too small cannot sustain complex cultural traits given copying errors. Regarding our extended criteria, there is only a single trait, a single lineage and a single fitness proxy, and so no functional dependence, diversification, recombination, exaptation or niche construction.

Subsequent models have added our extended criteria. Functional dependence is modelled as a sequence of discrete traits that must be acquired in order [41,51–54]. One resulting insight is that the cost of social and individual learning may increase as CCE proceeds, simply because there is more to learn, thus potentially slowing down CCE [51,52]. Some models allow diversification in multiple lineages and recombination across those lineages [38,53–55], often finding that recombination generates exponential increases in the number of cultural traits just like real-life human CCE [38,53]. The most sophisticated models are those of Kolodny and co-workers [53,56], which assume incrementally changing and recombining lineages branching off a main trait axis. However, no models have properly explored the possibility of multiple fitness proxies, nor the cultural exaptation and niche construction which may follow. Cultural fitness is often not explicitly modelled, beyond the assumption that traits increase in number over time; in order to model our final two extended criteria, we would need to assume multiple, changing fitness proxies that individual traits may fulfil.

## 5. Cumulative cultural evolution in non-human species

Electronic supplementary material, table S3 summarizes studies that have examined CCE (or precursors to CCE) in non-human species. Some field studies have claimed, on the basis of circumstantial evidence, that certain primates and corvids exhibit CCE [16,17,57], but there are few experimental studies capable of testing whether non-human animals meet our criteria. Some experiments suggest that chimpanzees may, under certain circumstances, switch from a relatively inefficient to a more efficient foraging or tool-use technique after observing others [14,58–60], consistent with our core criteria (i)–(iii), but do not examine the scope for repeated improvements (core criterion (iv)). Indeed, in a study using a three-stage sequential problem-solving task, chimpanzees and capuchin monkeys failed to build on learned behaviour to reach higher stages with more desirable food rewards [37]. Another study found that the performance of Guinea baboons on a spatial memory task improved across transmission chains when the pattern of stimuli seen by each baboon was derived from a previous individual's choices [15]. However, this experiment provided no direct opportunity for social learning (criterion (ii)).

Although research into non-human CCE has focused heavily on primates, the only two studies to provide evidence for all four core criteria involved birds [18,19] (see electronic supplementary material, table S2 for details). In Sasaki & Biro's study of homing pigeons [18], experimental conditions began with a single individual who learned a homing route over 12 trials. This individual then flew the route 12 times with a naive bird. Subsequently, across five ‘generations’, the most experienced bird of a pair was replaced with a naive individual. Replacement chains ended with shorter routes than control chains (lone birds or pairs flying the same route repeatedly), demonstrating all our core criteria: (i) behavioural change via individual or collective learning, (ii) social learning and (iii) improvements in route efficiency which (iv) were repeated over successive pair combinations.

In all experimental studies of non-human CCE, improvement can only occur in a single trait up to a single, fixed optimum. This contrasts with the open-endedness of much human CCE, which likely relies on some or all of our extended criteria. Nevertheless, research suggests that at least some non-human animals may exhibit simple forms of CCE, and raises the possibility that some facets of animal behaviour, from migration routes to tool use and the construction of elaborate structures, may stem, at least partly, from an incremental cultural history.

## 6. Human experiments

Electronic supplementary material, table S4 summarizes experimental studies of CCE in humans. These use a variety of tasks, including material (e.g. spaghetti towers), virtual (e.g. virtual fishing nets) and social (e.g. languages) artefacts, and different designs, including linear transmission chains and groups with or without replacement of members [61]. Most experiments in adults and children meet all our core criteria (electronic supplementary material, table S4). Demonstrations of our extended criteria are less common, but all have been observed at least once.

Functional dependence is shown in computer-based experiments where the invention of new tools is contingent on the presence of other tools. Derex & Boyd [62] provided participants with initial resources like stones that could be combined in sequence with other resources to produce compound tools like axes. McGuigan *et al*. [63] demonstrated similar functional dependence in children with a physical artefact, where longer stick tools were fashioned from shorter ones to extract rewards from a puzzle box.

Diversification is observed in studies using transmission chains. Caldwell & Millen [21] found that spaghetti tower and paper airplane designs gradually became more similar within chains than between chains. However, their analyses also suggest that chains became more similar across time, probably because inherently more successful designs were universally favoured. Evidence for long-term diversification comes from a study that implemented separate design trajectories each of which led to different optima [39]. Overall, electronic supplementary material, table S4 suggests that diversification is often possible in experiments but rarely formally analysed.

Recombination, cultural exaptation and cultural niche construction are seldom investigated experimentally, probably due to the difficulty of designing tasks that feature these more open-ended criteria. In the aforementioned study implementing multiple optima [39], recombination across lineages allowed novel combinations to be created. In another [62], participants could create tools with different functions (e.g. axes for cutting or pigments for decorating), which could be used across functional categories (e.g. axes could be used to crush berries to make paint) demonstrating cultural exaptation, and some functions could only be reached after certain traits had been accumulated, demonstrating cultural niche construction. Beyond these rare ‘proof-of-concept’ studies, the full scope and consequences of these extended criteria have yet to be fully explored in the laboratory.

## 7. Key questions

### (a) What distinguishes core and extended criteria?

Our review of the non-human literature suggests that while some species meet our core criteria, none show evidence of the extended criteria. While it is often claimed that CCE underpins human ecological success, perhaps one or all of the *extended* criteria are actually responsible for this. If so, it is instructive to ask what distinguishes the extended criteria from the core.

One possibility is that our core criteria involve the reduction of uncertainty and increase in learnability, while the extended criteria involve an increase in uncertainty and reduction in learnability. In Sasaki & Biro's [18] pigeon study, for example, which involves only our core criteria, initial uncertainty about the optimal route is reduced by repeated individual and social learning until chains reach the single most-efficient route. Once the optimum is reached, no uncertainty remains. Given that learning is a means of reducing uncertainty about the world, we can conversely view this in terms of ‘learnability’: there is either no change in the difficulty of learning successive routes, or perhaps an increase in learnability if beeline routes are easier to learn than more convoluted routes.

Our extended criteria, however, typically involve an increase in uncertainty and an attendant decrease in learnability. Functional dependence makes compound traits increasingly harder to learn, as prior steps (e.g. arithmetic) must be mastered before later steps (e.g. calculus) can be acquired [51]. Diversification and recombination result in an exponential increase in design space, vastly increasing the range of possible behavioural options available to learners. Cultural niche construction creates new fitness proxies, each of which represent entirely new design spaces. Our extended criteria, then, generate the ‘open-endedness’ that is characteristic of human CCE.

This distinction between uncertainty-reducing and uncertainty-increasing processes can be viewed in terms of information in the Shannon–Weaver entropy sense, where information is a measure of the number of states that a system can take. However, many have argued that information in this sense misses key features of biological systems [64,65]. A valuable future task would be to integrate CCE into explicitly evolutionary theories of information, such as those based on statistical decision theory [64].

In principle, ‘learnability’ can be operationalized by measuring the probability that a naive individual invents or discovers a trait on their own, or the time it takes to learn a trait. Our core criteria involve an increase or no change in this ‘learnability’ measure, while our extended criteria involve a decrease. This resembles Tennie *et al*.'s [11] notion of ‘zone of latent solutions' (ZLS), which encompasses behaviours that individuals ‘could easily invent on their own’ (p. 2 406). They argue, similarly to us, that only human culture exceeds this ZLS. However, we would see this as an outcome of our extended criteria rather than a criterion itself. We would also see learnability as a continuous measure rather than a discrete ‘zone’. A problem with both ‘learnability’ and the ZLS is that it is impractical to test learning in truly ‘naive’ individuals, especially humans. It is impossible to create a Robinson Crusoe-style experiment to test what a single individual can or cannot invent alone. Asocial conditions in experiments can be used, but people come into experiments already possessing huge amounts of culturally acquired knowledge. Moreover, experiments last a few hours at most, rather than an entire lifetime. Nevertheless, further development of this learnability criterion is recommended.

This distinction also links to debates over language evolution and cultural attraction. In experimental studies of language evolution [66], artificial languages become more easily learned via repeated transmission, to a point where they are maximally learnable and expressive. This also applies to cases of iterated learning where there is a single intuitive prior upon which chains converge [67]. Cultural attraction theorists, similarly, focus on cases where cultural representations converge on intuitive, easily learnable and reconstructible forms [22]. These would all be cases of our core criteria, entailing a reduction in uncertainty and increase in learnability. Cases of technological or scientific CCE, however, seem to entail our extended criteria given their open-endedness and decrease in learnability. Disagreement over the importance of CCE in human culture may arise due to confusion between what we are calling core and extended criteria: some cases of human CCE involve core criteria, others extended.

### (b) What is ‘fitness’ in cumulative cultural evolution?

Virtually, all definitions of CCE specify ‘improvement’ as a requirement of CCE, hence its inclusion in our core criteria. We suggested above that this involves an improvement in some measure of performance, which is a proxy of genetic and/or cultural fitness. Yet, the notion of fitness is under-theorized in the context of CCE. A core assumption of behavioural ecology is that behaviour evolves to maximize inclusive (direct and indirect genetic) fitness. In practice, fitness is typically assessed using measures such as reproductive success (number of surviving offspring) or indirect proxies of reproductive success such as food intake, energy efficiency or mating frequency. Purported cases of CCE in non-human species use such measures and proxies. Macaques may improve potato washing techniques to acquire more food or make food more digestible [16]. Pigeons may improve their flight efficiency to minimize energy expenditure during migration to nesting or feeding sites [18]. It is a reasonable assumption that increased food intake and energy efficiency translate into higher reproductive success and hence inclusive fitness.

Some cases of human CCE can be understood similarly, particularly in non-agricultural, non-market economies. For example, the spear-thrower and bow-and-arrow are projectile technologies that improve the rate of food acquisition for their users and users' families. In other cases, particularly in agricultural or industrialized societies, inclusive fitness benefits are harder to see. Does knowledge of quantum physics enhance the inclusive fitness of its bearers? Do smartphones enhance the inclusive fitness of their users? This links to broader debates within human behavioural ecology over fitness maximization in societies that have undergone the demographic transition to low fertility and mortality [68,69]. Proxies like monetary or material wealth, or social status, may be more appropriate measures for human CCE, given evidence that people in post-transition societies appear not to maximize fertility [69]. Knowledge of quantum physics provides employment, a salary and social status, without necessarily maximizing inclusive (genetic) fitness or reproductive success. Given this disconnect with inclusive fitness, it may be more appropriate to talk of ‘cultural fitness’, the degree to which a product of CCE maximizes indirect proxies such as wealth or social status. Gene–culture coevolution models suggest specific cases when genetic and cultural fitness might diverge [29,30], such as when wealth or status as indicators of whom to copy exhibit ‘runaway’ cultural selection [29] or when maladaptive practices are more visible than more effective alternatives [70].

A separate issue to whether the products of CCE enhance the fitness of their bearers relates to fitness benefits to innovators versus copiers. This applies to humans and non-human species. In a sense, CCE is a cooperative dilemma: innovators produce knowledge at some cost, while others can copy them at less cost. In principle, this informational collective action problem suffers the same challenges as non-informational collective action problems such as maintaining fishing stocks: free-riders may exploit the knowledge of innovators causing innovation to cease [71]. While social learning has been modelled as a cooperative (producer–scrounger) dilemma [72], this is seldom placed in a CCE context. Perhaps our extended criteria can only emerge when this collective action problem is solved via institutions such as patent systems or patronage [73] that ensure benefits to innovators. Alternatively, fitness benefits of CCE could accrue to groups rather than individuals, if groups with superior CCE due to group members freely sharing knowledge outcompete groups of informational free-riders with inferior CCE [74]. Further work should specify the dynamics of CCE within a multilevel selection framework and carefully delineate the fitness benefits and costs of different components of CCE to individuals and groups.

Finally, while our core criteria assume a single measure of performance, our final two extended criteria involve multiple measures and the creation of new measures. Perhaps another reason for human ecological success is not CCE *per se*, but the ability for CCE to modify fitness proxies, generating an open-ended dynamic without being tied tightly to inclusive fitness. Interestingly, Kaplan *et al*. [69] suggest that the failure of people in post-demographic transition societies to maximize inclusive fitness may *result* from CCE, due to the higher parental investment needed for children to acquire, via formal education, ever-accumulating cultural knowledge. Consequently, the modification of fitness proxies characterized in our extended criteria may be both a consequence of CCE and a facilitator of further CCE.

### (c) What socio-cognitive capacities underlie cumulative cultural evolution?

Ongoing empirical work has attempted to delineate the socio-cognitive capacities that underlie CCE, such as imitation, teaching or theory of mind [37,75,76], or demographic conditions such as partially connected populations [39,56], but no consensus has emerged. The evidence reviewed above suggests that CCE can emerge in the absence of ‘higher’ cognitive capacities [18]. Our multiple criteria suggest that it may be fruitless looking for a single cognitive capacity, or even suite of cognitive capacities, that underlie CCE, if CCE itself comprises multiple subcomponents. Different socio-cognitive capacities may underlie our core criteria and each extended criteria. Furthermore, it may be incorrect to treat cognition as a static, exogenous, species-specific factor that permits (or does not permit) CCE. The learned content of CCE may itself enhance cognitive capacities—a human example would be reading and writing, cultural inventions which seem to increase intelligence [77]. This may, in turn, facilitate further CCE, which further enhances cognition, in an ongoing coevolutionary dynamic. Whether this dynamic also applies to non-human species and the nature of this coevolution (e.g. whether genetic changes are involved) are worthy of further study.

## 8. Conclusion

We have attempted to highlight the multiple senses in which CCE is used in the literature. We have identified a set of core criteria that seem essential for CCE: the introduction of behavioural novelty or modification, the transmission of behaviour via social learning, the improvement in genetic and/or cultural fitness or fitness proxies as a result of the learned behaviour and the repeated transmission and improvement of the behaviour over time. These criteria are central to the original formulation of CCE [2,3] and the most influential models of CCE [50]. We also specify a set of extended criteria—diversification, recombination, exaptation and niche construction—that seem to be involved in many of the paradigmatic cases of human CCE cited in the literature, but do not appear as yet to have been observed in non-human species.

We suggest that treating CCE as unitary phenomenon, and especially as a rubicon between human and non-human species, is unhelpful. Researchers should be explicit about which criteria they are testing. Although CCE is commonly cited as the key to human ecological success, we suspect that only our extended criteria actually underlie this success. As shown in electronic supplementary material, table S3, our core criteria have been demonstrated in non-human species that would not normally be attributed human-like levels of ecological dominance. This does not make such findings any less interesting, and indeed, linking such phenomena to human success may unnecessarily detract from their importance. Similarly, seeking a single set of socio-cognitive capacities that underlie CCE may benefit from specifying the precise CCE criteria being tested, given that different cognitive capacities may underlie different core and extended criteria, and different species may achieve the same criteria with different cognitive mechanisms. Finally, we suspect that much will be gained by a deeper consideration of the informational basis and consequences of CCE processes, the fitness dynamics of CCE, such as the modification and creation of cultural fitness proxies, and the dynamics of CCE as a cooperative dilemma within a multilevel selection framework.

## Supplementary Material

Tables S1 - S4

## Data accessibility

This article has no additional data.
