# Supplementary material for: What is cumulative cultural evolution?
Source: Proc Biol Sci. 2018 Jun 13;285(1880):20180712. doi: 10.1098/rspb.2018.0712 (PMC6015846; doi:10.1098/rspb.2018.0712)
Supplement: Tables S1 - S4 [file rspb20180712supp1.pdf]

Supplementary Material for

Mesoudi, A. and Thornton, A. (2018) What is cumulative cultural evolution?
Proceedings of the Royal Society B 20180712

Contents:

Table S1: Definitions of cumulative cultural evolution
Table S2: Models of cumulative cultural evolution that feature our core and extended criteria
Table S3: Evidence for our core and extended criteria in non-human animal studies of cumulative cultural evolution
Table S4: Human experimental studies of cumulative cultural evolution that feature our core and extended criteria

Table S1: Definitions of cumulative cultural evolution

"By cumulative culture we mean a cumulative improvement in the adaptiveness of a cultural trait, although the term can also describe an increase in the number of adaptive cultural traits." (Aoki & Feldman 2014, p.14)
CCE occurs when "the achievements of one pattern of behavior form the basis for the selection of a modified and better-adapted descendant pattern" (Avital and Jablonka, 2000, p.94)
"...social learning allows learned improvements to accumulate from one generation to the next. When learning in small steps is less costly per unit improvement in fitness than learning in large steps, the cumulative learning over many generations can increase average fitness." (Boyd & Richerson 1995, p.134)
"The term CCE [cumulative cultural evolution] is used to describe the way that knowledge accumulates in human populations over time, such that each generation makes use of behaviours and artefacts invented by previous generations, which they would be unlikely to have been able to invent by themselves" (Caldwell & Millen 2008, p.165)
"The term "cumulative cultural evolution" refers to situations in which social transmission allows for successive improvements to performance over generations of learners, generated by the accumulation of modifications to the transmitted behaviors" (Caldwell & Millen 2010, p.123)
"Cumulative cultural evolution is often defined as a specific case of cultural evolution, where such changes exhibit a directional trend, typically representing improvements upon, or elaborations of, earlier alternatives." (Caldwell, Renner & Atkinson 2017, p.2)
"Cumulative culture [is] cultural information too complex for a single individual to devise in a one [sic] generation." (Chudek & Henrich 2011, p.219)
"In humans, this iterated learning procedure leads to three fundamental properties of human cumulative culture: (i) a progressive increase in performance; (ii) the emergence of systematic structure and (iii) lineage specificity, with different kinds of structure emerging in different chains" (Claidiere et al. 2014, p.1)
"we describe as *accumulation*, the addition of knowledge or behaviour patterns to the behavioural repertoire of an individual or population..., and restrict use of the phrase *cumulative culture* to the modification, over multiple transmission episodes, of cultural traits (behavioural patterns transmitted through social learning) resulting in an increase in the complexity or efficiency of those traits." (Dean et al. 2014, p.4)
""cumulative culture" — the extensive accumulation of knowledge, and iterative improvements in technology, over time" (Dean et al. 2012, p.1114)
"To make the process of cultural accumulation realistic, we specified that innovations were contingent upon earlier discoveries and resulted from incremental improvement or recombination of different traits" (Derex & Boyd 2016, p.2982)
"By ''cumulative culture'' we mean the capability to accumulate knowledge, and for iterative improvements in technology, over multiple generations. The capacity of human culture to amass ever more effective solutions through repeated bouts of innovation and social transmission, leading to the evolution of technology that no individual could alone invent, has been described as like a ''ratchet''" (Ehn & Laland 2012, p.103)
"A striking aspect of humans is their ability to shape and accumulate cultural information over generations, to an extent that could not be achieved by a single generation...That is, human culture is *cumulative*." (Enquist et al. 2008, p.46)
"Dependencies refer to relationships between elements, such that the presence of one cultural element affects the likelihood that another element appears or disappears. Dependencies between cultural elements seem to us to constitute the core of what cumulative culture is about. Other conceptualizations of cumulative culture in the literature seem to be special cases that are too limited." (Enquist et al. 2011, p.412)
"[human culture is] *cumulative*, i.e., it typically increases over generations to levels that no single generation could achieve" (Ghirlanda & Enquist 2007, p.1)
"*cumulative cultural evolution* [is] the point at which culturally transmitted information begins to accumulate over generations, such that tools and know-how get increasingly better fit to the local environments–this is the "ratchet effect"" (Henrich 2015, p.56-57)
"Three important characteristics of cumulative technological evolution are: (i) diversification of tool design; (ii) cumulative change to tool lineages; and (iii) faithful transmission of tool design through social learning" (Hunt & Gray 2003, p.867)

"*Cumulative* cultural evolution refers to the presence of traits that have been gradually modified and built upon over successive generations such that they are beyond the capabilities of a single individual to invent in a single lifetime." (Kempe & Mesoudi 2014a, p.319)
“cumulative culture [is] defined as cultural traits that are dependent on other cultural traits...Cumulative culture is often characterised by the presence of traits that are too complex to have been invented by a single individual, instead having accumulated over multiple generations” (Kempe & Mesoudi 2014b, p.285)
“this process [of iterated language learning] is cumulative and is not considered to arise from the explicit intentions of the individuals involved. Rather, this type of cultural evolution is an ‘‘invisible hand’’ process leading to phenomena that are the result of human action but are not intentional artifacts” (Kirby et al. 2008, p.10681)<br><br>"Note that this adaptation [in artificial languages] is cumulative with respect to learnability and structure but not with respect to expressivity: cumulative adaptation does not suggest that the languages necessarily become more functional with respect to communication." (Kirby et al. 2008, p.10685)
"Recent human culture is characterized by the *ratchet effect* (Tomasello, 1994), or an increase in the complexity or efficiency of technology over time." (Laland 2004, p.10)
"human culture appears unique in that it is cumulative, i.e. human cultural traits increase in diversity and complexity over time." (Lewis & Laland 2012, p.2171)
"Humans possess a complex cumulative culture manifest in the presence of knowledge, artefacts and technology, and complex cultural institutions. These traits are not typically produced in a single step, but are produced by small, incremental changes over time, with refinements of existing knowledge and technology, a process known as ‘ratcheting’" (Lewis & Laland 2012, p.2171)
"One of the hallmarks of human culture is that it is cumulative. Beneficial innovations are accumulated and combined over time resulting in knowledge and technology that could not have been invented by a single individual on their own" (Mesoudi 2011, p.1)
“Cumulative cultural evolution [is] the preservation of cultural traits over successive generations such that individuals acquire knowledge that exceeds what any single individual could invent alone.” (Mesoudi 2016, p.18)
"The processes of cumulative cultural evolution result in technologies and techniques that no single individual could recreate in their lifetime, and do not require its beneficiaries to understand how and why they work" (Muthukrishna & Henrich 2016, p.2)
“we modeled the process of cultural accumulation of technology, which we defined as adding new actions to existing ones to create new functional combinations” (Pradhan et al. 2012, p.180)<br><br>“The paradigmatic case of ratcheting is when an individual adds an existing technique used in a different context, or an entirely novel technique, to an existing technique, and integrates them functionally.” (Pradhan et al. 2012, p.181)
"culture can accumulate progressively, or ‘ratchet’, over generations — a process referred to as Cumulative Cultural Evolution (CCE)...CCE can allow groups to develop increasingly complex knowledge and skills over time, beyond the capacities of a single individual." (Sasaki & Biro 2017, p.2)
“We...define CC [cumulative culture] as a modification (change in the sequence or form of behavioral elements) of a cultural trait (i.e., acquired via social learning) that enhances its complexity, efficiency, security, or convenience.” (Schofield et al. 2017, p.2)
"a key feature of uniquely human cultural products and practices is that they are cumulative. One generation does things in a certain way, and the next generation then does them in that same way — except that perhaps they add some modification or improvement. The generation after that then learns the modified or improved version, which then persists across generations until further changes are made. Human cultural transmission is thus characterized by the so-called ‘ratchet effect’, in which modifications and improvements stay in the population fairly readily (with relatively little loss or backward slippage) until further changes ratchet things up again." (Tennie et al. 2009, p.2405)
"the cultural traditions and artifacts of human beings accumulate modifications over time in a way that those of other animal species do not — so-called cumulative cultural evolution. Basically none of the most complex human artifacts or social practices — including tool industries, symbolic communication, and social institutions — were invented once and for all at a single moment by any one individual or group of individuals. Rather, what happened was that some individual or group of individuals first invented a primitive version of the artifact or practice, and then some later user or users made a modification, an “improvement,” that others then adopted perhaps without

change for many generations, at which point some other individual or group of individuals made another modification, which was then learned and used by others, and so on over historical time in what has sometimes been dubbed “the ratchet effect”...The process of cumulative cultural evolution requires not only creative invention but also, and just as importantly, faithful social transmission that can work as a ratchet to prevent slippage backward — so that the newly invented artifact or practice preserves its new and improved form at least somewhat faithfully until a further modification or improvement comes along." (Tomasello 1999, p.5)
“This process of cumulative culture [is] the successive addition or blending of new innovations and old, and their social spread within populations and across generations” (Vale et al. 2017, p.635)
“...*cumulative cultural change*, which describes cultural change across generations that allows individuals to achieve phenotypes that they could not achieve within their lifetime through asocial learning.” (van der Post et al. 2017, p.1)
"many argue that humans are still unique in their capacity for cumulative cultural evolution, with successive generations building on earlier achievements" (Yamamoto et al. 2013, p.1)
"Human culture accumulates and increases in complexity over time, building on the achievements of previous generations in a ratchet-like manner. This phenomenon, termed cumulative culture, generates ever-more efficient tools, technologies and social structures" (Zwirner & Thornton 2015, p.1)

References for Table S1

Aoki K, Feldman MW. 2014 Evolution of learning strategies in temporally and spatially variable environments: A review of theory. Theoretical Population Biology 91, 3–19.

Avital E, Jablonka E. 2000 Animal traditions: Behavioural inheritance in evolution. Cambridge: Cambridge University Press.

Boyd R, Richerson PJ. 1995 Why does culture increase human adaptability? Ethology and Sociobiology 16, 125–143.

Caldwell CA, Millen AE. 2008 Experimental models for testing hypotheses about cumulative cultural evolution. Evolution and Human Behavior 29, 165–171.

Caldwell CA, Millen AE. 2010 Human cumulative culture in the laboratory: Effects of (micro) population size. Learning & Behavior 38, 310–318.

Caldwell CA, Renner E, Atkinson M. 2017 Human teaching and cumulative cultural evolution. Review of Philosophy and Psychology.

Chudek M, Henrich J. 2011 Culture–gene coevolution, norm-psychology and the emergence of human prosociality. Trends in Cognitive Sciences 15, 218–226.

Claidière N, Smith K, Kirby S, Fagot J. 2014 Cultural evolution of systematically structured behaviour in a non-human primate. Proceedings of the Royal Society of London B: Biological Sciences 281, 20141541.

Dean LG, Kendal RL, Schapiro SJ, Thierry B, Laland KN. 2012 Identification of the social and cognitive processes underlying human cumulative culture. Science 335, 1114–1118.

Dean LG, Vale GL, Laland KN, Flynn E, Kendal RL. 2014 Human cumulative culture: a comparative perspective. Biological Reviews 89, 284–301.

Derex M, Boyd R. 2016 Partial connectivity increases cultural accumulation within groups. Proceedings of the National Academy of Sciences 113, 2982–2987.

Ehn M, Laland K. 2012 Adaptive strategies for cumulative cultural learning. Journal of Theoretical Biology 301, 103-111.

Enquist M, Ghirlanda S, Eriksson K. 2011 Modelling the evolution and diversity of cumulative culture. Philosophical Transactions of the Royal Society B 366, 412–423.

Enquist M, Ghirlanda S, Jarrick A, Wachtmeister CA. 2008 Why does human culture increase exponentially? Theoretical Population Biology 74, 46–55.

Ghirlanda S, Enquist M. 2007 Cumulative culture and explosive demographic transitions. Quality and Quantity 41, 591–600.

Henrich J. 2015 The secret of our success. Princeton University Press.

Hunt GR, Gray RD. 2003 Diversification and cumulative evolution in New Caledonian crow tool manufacture. Proceedings of the Royal Society of London B 270, 867–874.

Kempe M, Mesoudi A. 2014 An experimental demonstration of the effect of group size on cultural accumulation. Evolution and Human Behavior 35, 285–290.

Kempe M, Mesoudi A. 2014 Experimental and theoretical models of human cultural evolution. Wiley Interdisciplinary Reviews: Cognitive Science 5, 317–326.

Kirby S, Cornish H, Smith K. 2008 Cumulative cultural evolution in the laboratory: An experimental approach to the origins of structure in human language. Proceedings of the National Academy of Sciences 105, 10681–10686.

Laland KN. 2004 Social learning strategies. Animal Learning & Behavior 32, 4–14.
Lewis HM, Laland KN. 2012 Transmission fidelity is the key to the build-up of cumulative culture. Philosophical Transactions of the Royal Society B 367, 2171–2180.
Mesoudi A. 2011 Cultural evolution. Chicago, IL: Univ. Chicago Press.
Mesoudi A. 2016 Cultural evolution: Integrating psychology, evolution and culture. Current Opinion in Psychology 7, 17–22.
Muthukrishna M, Henrich J. 2016 Innovation in the collective brain. Philosophical Transactions of the Royal Society B 371, 20150192.
Pradhan GR, Tennie C, van Schaik CP. 2012 Social organization and the evolution of cumulative technology in apes and hominins. Journal of Human Evolution 63, 180–190.
Sasaki T, Biro D. 2017 Cumulative culture can emerge from collective intelligence in animal groups. Nature Communications 8, 15049.
Schofield DP, McGrew WC, Takahashi A, Hirata S. 2017 Cumulative culture in nonhumans: overlooked findings from Japanese monkeys? Primates 59, 113–122.
Tennie C, Call J, Tomasello M. 2009 Ratcheting up the ratchet: on the evolution of cumulative culture. Philosophical Transactions of the Royal Society B 364, 2405–2415.
Tomasello M. 1999 The cultural origins of human cognition. Cambridge, MA: Harvard Univ. Press.
Vale GL, Davis SJ, Lambeth SP, Schapiro SJ, Whiten A. 2017 Acquisition of a socially learned tool use sequence in chimpanzees. Evolution and Human Behavior 38, 635–644.
van der Post DJ, Franz M, Laland KN. 2017 The evolution of social learning mechanisms and cultural phenomena in group foragers. BMC Evolutionary Biology 17, 49.
Yamamoto S, Humle T, Tanaka M. 2013 Basis for cumulative cultural evolution in chimpanzees. PLOS One 8, e55768.
Zwirner E, Thornton A. 2015 Cognitive requirements of cumulative culture. Scientific Reports 5, 16781.

Table S2: Models of cumulative cultural evolution that feature our core and extended criteria

Model	Innovation	Social learning	Improvement	Repeated improvement	Functional dependence	Diversification	Recombination	Cultural exaptation	Cultural niche construction
Henrich 2004 (also Powell et al. 2009)	Yes - via "lucky guesses and errors", new traits are drawn randomly from a gumbel distribution	Yes – indirectly biased copying of the individual with the highest skill	Yes - via innovation that results in higher skill than that of the copied individual	Yes - up to the limit given by population size and transmission accuracy	No - a single trait is improved	No - single lineage	No	No – only a single fitness proxy (z)	No
Mesoudi 2011	Yes – new traits drawn randomly from an exponential distribution	Yes - either directly biased copying of best traits, or indirectly biased copying of best individual, or random copying	Yes – via directly or indirectly biased social learning of best innovations or individuals	Yes - up to the limit given by population size and lifetime learning capacity	Yes – multiple traits that must be learned in sequence	No – single lineage	No	No – only a single fitness proxy (z)	No
Enquist et al. 2011	Yes – in terms of the appearance of new traits	Yes – implicitly in that invented traits persist	Partly – only in terms of number of traits; fitness is not explicitly modelled	Partly – traits can increase in number indefinitely	Yes – traits may only appear when prior traits are present	Yes – multiple lineages	Yes – different traits from different lineages can be combined	No – fitness is not explicitly modelled	No – fitness is not explicitly modelled
Lewis & Laland 2012	Yes – drawn randomly from a uniform distribution	Yes – implicitly in that invented traits persist; also payoff biased such that low-utility traits are more likely to be lost	Yes – increase in 'trait utility' via payoff-biased social learning	Yes – up to the limit given by transmission accuracy	Yes – multiple traits	Yes – multiple lineages	Yes – different traits from different lineages can be combined	No – only a single fitness proxy ('utility')	No
Ehn & Laland 2012	Yes – invention of new traits at successive refinement levels	Yes – copying of randomly selected individuals	Yes – each refinement level increases fitness	Yes – refinement can continue indefinitely up to the limit set by accuracy of learning	Yes – each refinement level is dependent on previous levels	No – single lineage	No	No – only a single fitness measure	No
Pradhan, Tennie & van Schaik	Yes – small probability of	Yes – individuals learn	Partly – refinement	Partly – up to a predetermined	Yes – each technology level	No – single lineage	No	No – fitness not explicitly	No

2012	innovating a new cultural trait	from neighbours in social networks	levels are assumed implicitly to represent improvements, but fitness not explicitly modelled	upper limit (technology level 4), and dependent on social learning opportunity / accuracy	is dependent on previous levels			modelled	
Kobayashi & Aoki 2012	Yes - via “lucky guesses and errors”, new traits are drawn randomly from a gumbel distribution	Yes – indirectly biased copying of the individual with the highest skill	Yes - via innovation that results in higher skill than that of the copied individual	Yes - up to the limit given by number of acquaintances and innovation rate	No - a single trait is improved	No - single lineage	No	No – only a single fitness proxy (z)	No
Kempe, Lycett & Mesoudi 2014	Yes – a new trait is discovered with a fixed porbability	Yes – from *n* randomly selected demonstrators	Partly – only in terms of number of traits; fitness is not explicitly modelled	Partly – traits can increase in number indefinitely, dependent on transmission accuracy and number of demonstrators	Yes – each trait is dependent on a previous trait being known	No - single lineage	No	No – fitness is not explicitly modelled	No
Nakahashi 2014	Yes - via random modification of acquired cultural traits	Yes – indirectly biased copying of the individual with the highest skill	Yes - via innovation that results in higher skill than that of the copied individual	Yes - up to the limit given by transmission accuracy and strength of direct bias	No - a single trait is improved	No - single lineage	No	No – only a single fitness proxy (z)	No
Kolodny et al. 2015, 2016; Creanza et al. 2017	Yes, via ‘lucky leaps’, incremental elaborations or recombination	Yes – inter-generational persistence is assumed unless traits are lost	Partly – only in terms of number of traits (tools); fitness is not explicitly modelled	Partly – traits can increase in number indefinitely	Yes – within toolkit lineages, each trait is dependent on the previous traits	Yes – multiple lineages branch off the main-axis tool lineage	Yes – main-axis traits can combine with existing tools	No – different lineages are stated to be maximising different proxies, but fitness is not explicitly modelled.	No - new lineages are assumed to be maximising novel proxies, but fitness is not explicitly modelled.
Derex et al. 2018	Yes – new traits are added with a fixed	Yes – individuals copy the most	Partly – more complex traits are assumed to	Partly – traits can increase in complexity	Yes – traits must be added in sequence to	Yes – multiple lineages are possible both	Yes – either within groups or (via migration)	No – only a single fitness proxy	No

	probability	complex trait from others in their group	be more fit, but fitness not explicitly modelled	indefinitely	increase in complexity level	within and between groups	between groups	(complexity)	

References for Table S2

Creanza N, Kolodny O, Feldman MW. 2017 Greater than the sum of its parts? Modelling population contact and interaction of cultural repertoires. Journal of The Royal Society Interface 14, 20170171.

Derex M, Perreault C, Boyd R. 2018 Divide and conquer: intermediate levels of population fragmentation maximize cultural accumulation. Philosophical Transactions of the Royal Society B 373, 20170062.

Ehn M, Laland K. 2012 Adaptive strategies for cumulative cultural learning. Journal of Theoretical Biology 301, 103-111.

Enquist M, Ghirlanda S, Eriksson K. 2011 Modelling the evolution and diversity of cumulative culture. Philosophical Transactions of the Royal Society B 366, 412–423.

Henrich J. 2004 Demography and cultural evolution. American Antiquity 69, 197–214.

Kempe M, Lycett SJ, Mesoudi A. 2014 From cultural traditions to cumulative culture: Parameterizing the differences between human and nonhuman culture. Journal of Theoretical Biology 359, 29–36.

Kobayashi Y, Aoki K. 2012 Innovativeness, population size and cumulative cultural evolution. Theoretical Population Biology 82, 38–47.

Kolodny O, Creanza N, Feldman MW. 2015 Evolution in leaps: The punctuated accumulation and loss of cultural innovations. Proceedings of the National Academy of Sciences 112, E6762–E6769.

Kolodny O, Creanza N, Feldman MW. 2016 Game-changing innovations: how culture can change the parameters of its own evolution and induce abrupt cultural shifts. PLOS Computational Biology 12, e1005302.

Lewis HM, Laland KN. 2012 Transmission fidelity is the key to the build-up of cumulative culture. Philosophical Transactions of the Royal Society B 367, 2171–2180.

Mesoudi A. 2011 Variable cultural acquisition costs constrain cumulative cultural evolution. PLOS One 6, e18239.

Nakahashi W. 2014 The effect of cultural interaction on cumulative cultural evolution. Journal of Theoretical Biology 352, 6–15.

Powell A, Shennan SJ, Thomas MG. 2009 Late Pleistocene demography and the appearance of modern human behavior. Science 324, 1298–1301.

Pradhan GR, Tennie C, van Schaik CP. 2012 Social organization and the evolution of cumulative technology in apes and hominins. Journal of Human Evolution 63, 180–190.

Table S3: Evidence for our core and extended criteria in non-human animal studies of cumulative cultural evolution

Study/ species	Task	Innovation	Social learning	Improvement	Repeated improvement	Functional dependence	Diversification	Recombination	Cultural exaptation	Cultural niche construction
Sasaki & Biro 2017 – homing pigeons (Experimental)	Learning direct homing routes	Yes – individuals can modify homing routes	Yes – individuals within pairs may learn from one another	Yes	Yes – improvement in a single trait, but only up to the bee-line optimum	No – only a single trait (route)	Partly – exogenously imposed by having multiple chains which initially diverged, but eventually converged	No	No	No
Fehér et al. 2009 – zebra finches (Experimental)	Song learning	Yes – individuals introduce modifications to learned song	Yes – vocal learning	Yes – but a product of experimental design: reversion to wild type song from aberrant, unstructured song of isolated males. Evidence for improvement is based on the assumption that songs more similar to the wild type should be preferred by females, therefore increasing reproductive success.	Yes – Improvement in a single trait (song learning) to a fixed maximum beyond which there is no potential for improvement: aberrant song of untutored males improves through a transmission chain until it resembles wild type	No – single trait (song)	Maybe – some indication of differences between transmission chains, but not formally analysed.	No	No	No
Claidière et al. 2014 – Guinea baboons (Experimental)	Transmission chain study involving memorising patterns of blocks on a touch screen	Yes – In the context of the experiment, errors may be equivalent to 'mutation'	Yes – But imposed by experimental design as the pattern of choices of each individual was used by experimenters to generate the patterns shown to the next individual in the transmission chain	Partly – No opportunities for observational learning. Social transmission of information imposed by experimental design as the pattern of choices of each individual was used by experimenters to generate the patterns shown to	Yes – Improvements in individual performance (correctly remembered blocks) across transmission chains	No – single trait (performance)	Yes – Emergence of different patterns in different transmission chains	No	No	No

				the next individual in the transmission chain						
Dean et al. 2012 – chimpanzees and capuchin monkeys, compared to human children (Experimental)	Sequential problem solving: three-stage puzzlebox providing increasingly valuable rewards#	Yes – individuals discovered techniques to access food	Partly – Some evidence for social learning among chimps, but only in Stage 1	No – Animals typically failed to obtain more desirable rewards (NB there is no evidence that more desirable rewards had greater nutritional value)	No – little evidence that chimps or capuchins could socially learn to reach progressively higher stages	Yes - but imposed by experimental design: in scaffolded condition, higher stages were locked until the previous stage was completed	No	No	No	No
Marshall-Pescini & Whiten 2008 – chimpanzees (Experimental)	Foraging puzzle-box could be opened in two ways: more complex (“probing”) method yielded greater rewards than simple “dipping” method	Yes – some individuals discovered techniques to access food	Partly – social learning from human demonstrator, but only of dipping method	No – Asocial learning of probing by one individual only; no social learning of probing	No – By experimental design, only two techniques were possible	Yes – but imposed by experimental design	No	No	No	No
Price et al. 2009 – chimpanzees (Experimental)	Making multi-component tools	Yes – some individuals learned to use and combine tools	Yes – individuals that saw video demonstrations were more likely to learn to make multi-component tools (but some individuals also learned in the absence of demonstration, so social learning may not be necessary)	Yes – in some conditions, food rewards could only be accessed by putting together components to make a longer tool	No – No potential for repeated improvements in the experiment	No – modification of a stick tool design (joining short tools to form a long tool), rather than the production of a new trait.	No	No	No	No
Yamamoto et al	Using tools to	Yes – individuals	Yes – social	Yes –	No –	No – learning	No	No	No	No

2013 – chimpanzees (Experimental)	obtain juice	spontaneously developed “sucking” or “dipping” technique	learning of sucking technique from conspecifics or human experimenters	Some individuals switched to a more effective technique after observing demonstrators (sucking)	No potential for repeated improvements in the experiment	the more efficient technique is not functionally dependent on having learned the inefficient technique				
Vale et al 2017 – chimpanzees (Experimental)	Using tools to obtain juice	Yes – individuals developed various task-oriented behaviours	Yes – chimps in seeded groups (but not unseeded groups) learned to use a tool as a straw	Yes – Learning a more efficient technique following observation of a trained demonstrator (though some individuals also learned to modify tools in the absence of a demonstrator)	No – No potential for repeated improvements in the experiment	Yes – but imposed by experimental design: modification of one tool produced a more efficient tool with different affordances. Some evidence that experience with simple tools was needed to learn behavioural sequences used for more complex tools.	No	No	No	No
Davis et al 2016 – chimpanzees (Experimental)	Manipulating a puzzle-box to extract tokens	Yes – e.g. one untrained chimp discovered the efficient method	Yes – chimps with access to social information changed their behaviour	Yes – Some individuals, forced by experimental design to use a highly inefficient method switched to a more efficient (faster) method after observing demonstrators	No – No potential for repeated improvements in the experiment	No	No	No – Some individuals combined elements of different techniques, but these were all behaviours performed within the context of the same task. No recombination of behaviours across lineages.	No	No
Kendal et al 2009 – nine-spined sticklebacks	Choosing between rich or poor foraging	Yes – individuals could learn through personal experience	Yes – individuals switch to the rich patch after	Yes – switching to the rich patch provides foraging returns	No – No potential for repeated improvements in	No	No	No	No	No

(experimental)	patches		observing demonstrators		the experiment					
Schofield et al 2017 – Japanese macaques (Observational)	Washing sweet potatoes and wheat	Yes – long-term field studies report emergence of novel behaviours like sweet-potato washing	Partly – descriptive evidence suggests novel behaviours spread from innovators through social networks	No – Authors suggest changes to behaviour may provide benefits (e.g. “Cleaner foodstuff reduces wear on teeth from sand”) but no empirical evidence	No – No empirical evidence but authors argue behavioural changes may generate improved foraging efficiency	No – But novel behaviours (e.g. digging pools to wash food) argued to build on previous socially learned behaviours	No	No	No	No – But verbal argument suggests initial innovation created novel conditions for later changes
Hunt & Gray 2003 New Caledonian crows (Observational)	Tool manufacture	No – Assumed, but not tested	No – Assumed, but not tested	No – No evidence that different tools differ in their efficacy	No – Assumed on the basis of circumstantial evidence, but not tested	No – But multi-step pandanus tools are suggested (on the basis of circumstantial evidence) to be cultural descendants of simpler, single-step tools	No	No	No	No
Filatova et al. 2013 – Killer whales (Observational)	Vocal dialects	Yes – changes in structure of songs	Yes – evidence for vocal learning (e.g. Crance et al. 2014)	No – Vocalisations do not become “better” in any functional sense. There may be cultural change (e,g, through processes analogous to drift), but not CCE according to our definition.	No – But some evidence for repeated change	No	Yes – Diversification of song lineages	Yes – some evidence for recombination of song components [e.g. Crance et al. 2014]	No	No – but agent-based models suggest vocal cultures in sperm whales may influence social structure and generate multi-level societies [Cantor et al. 2015]
Boesch 2003; Sanz et al. 2010 and others – wild chimpanzees (Observational)	Tool use and manufacture	Partly – some evidence for innovative tool-related behaviour	Yes – Descriptive evidence and network-based diffusion analyses suggest a role for social learning in the development of tool use	No – No direct evidence that supposedly complex tools are more effective than simpler tools	No – But arguments based on circumstantial evidence suggest some forms of tool use (e.g hammer and anvils for nut-cracking) and tool-kits are the	No – Argued by some authors, but no empirical evidence	No	No	No	No

					products of repeated instances of cultural change					
Perry et al. 2011 – wild capuchin monkeys (Observational)	Social conventions	Yes – individuals perform novel social behaviours	Partly – some evidence for transmission through social networks	No – no evidence that new behaviours provide fitness benefits	No	No	No	No	No	No

References for Table S3

Boesch C. 2003 Is culture a golden barrier between human and chimpanzee? Evolutionary Anthropology 12, 82–91.

Cantor M, Shoemaker LG, Cabral RB, Flores CO, Varga M, Whitehead H. 2015 Multilevel animal societies can emerge from cultural transmission. Nature Communications 6, 8091.

Claidière N, Smith K, Kirby S, Fagot J. 2014 Cultural evolution of systematically structured behaviour in a non-human primate. Proceedings of the Royal Society of London B 281, 20141541.

Crance JL, Bowles AE, Garver A. 2014 Evidence for vocal learning in juvenile male killer whales, Orcinus orca, from an adventitious cross-socializing experiment. Journal of Experimental Biology 217, 1229–1237.

Davis SJ, Vale GL, Schapiro SJ, Lambeth SP, Whiten A. 2016 Foundations of cumulative culture in apes. Scientific Reports 6, srep35953.

Dean LG, Kendal RL, Schapiro SJ, Thierry B, Laland KN. 2012 Identification of the social and cognitive processes underlying human cumulative culture. Science 335, 1114–1118.

Feher O, Wang H, Saar S, Mitra PP, Tchernichovski O. 2009 De novo establishment of wild-type song culture in the zebra finch. Nature 459, 564–568.

Filatova OA, Burdin AM, Hoyt E. 2013 Is killer whale dialect evolution random? Behavioural Processes 99, 34–41.

Hunt GR, Gray RD. 2003 Diversification and cumulative evolution in New Caledonian crow tool manufacture. Proceedings of the Royal Society of London B 270, 867–874.

Kendal JR, Rendell L, Pike TW, Laland KN. 2009 Nine-spined sticklebacks deploy a hill-climbing social learning strategy. Behavioral Ecology 20, 238–244.

Marshall-Pescini S, Whiten A. 2008 Chimpanzees (Pan troglodytes) and the question of cumulative culture. Animal Cognition 11, 449–456.

Perry S. 2011 Social traditions and social learning in capuchin monkeys (Cebus). Philosophical Transactions of the Royal Society B 366, 988–996.

Price EE, Lambeth SP, Schapiro SJ, Whiten A. 2009 A potent effect of observational learning on chimpanzee tool construction. Proceedings of the Royal Society of London B 276, 3377–3383.

Sanz CM, Schoning C, Morgan DB. 2010 Chimpanzees prey on army ants with specialized tool set. American Journal of Primatology 72, 17–24.

Sasaki T, Biro D. 2017 Cumulative culture can emerge from collective intelligence in animal groups. Nature Communications 8, 15049.

Schofield DP, McGrew WC, Takahashi A, Hirata S. 2018 Cumulative culture in nonhumans: overlooked findings from Japanese monkeys? Primates 59, 113–122.

Vale GL, Davis SJ, Lambeth SP, Schapiro SJ, Whiten A. 2017 Acquisition of a socially learned tool use sequence in chimpanzees. Evolution and Human Behavior 38, 635–644.

Yamamoto S, Humle T, Tanaka M. 2013 Basis for cumulative cultural evolution in chimpanzees. PLOS One 8, e55768.

Table S4: Human experimental studies of cumulative cultural evolution that feature our core and extended criteria

Study / species	Task	Innovation	Social learning	Improvement	Repeated improvement	Functional dependence	Diversification	Recombination	Cultural exaptation	Cultural niche construction
Caldwell & Millen 2008	Spaghetti tower and paper airplane manufacture	Yes – Changes in artefacts design	Yes - Observation of experienced participants by naïve participants	Yes – Towers got higher and planes flew farther	Yes – Improvements occurred over multiple generations	No	Yes – Artefacts were more similar within chains than between chains	No	No	No
Mesoudi 2008	Virtual arrowhead design	Yes – Changes in arrowhead design	Yes – Pay-off biased social learning	Yes – Arrowheads scores increased	Yes – Improvements occurred over multiple periods	No	Diversification was possible but not formally analysed	No	No	No
Kirby, Cornish & Smith 2008	Iterated learning of artificial languages	Yes – Changes in words and structures	Yes - Participants were trained on the output of the previous participant	Yes – Increases in learnability of languages	Yes – Increases in learnability occurred over time	No	Diversification was possible but not formally analysed	No	No	No
Beppu & Griffith 2009	Iterated learning of a one-dimensional function relating two variables	Yes - Variation was introduced through learning errors	Yes - Participants were trained on the output of the previous participant	No	No	No	Yes – Some chains converged to a positive linear relationship and one converged to a negative linear relationship	No	No	No
Wisdom & Goldstone 2010	Multi-dimensional problem-solving game (team composition with linear with linear and interactive effects on score)	Yes – Changes in team composition	Yes – participants were provided with the choices of their other group members	Yes – Increase in team performance	Yes – Improvements occurred over multiple periods	No	Diversification was possible but not formally analysed	No	No	No
Derex, Godelle & Raymond 2013	Virtual fishing net production	Yes – Changes in fishing net design	Yes – Pay-off biased social learning	Yes – Fishing nets scores increased	Yes – Improvements occurred over multiple periods	No	Yes – Fishing nets similarity was higher within-group than between-group	No	No	No
Wasielewski 2014	Weight bearing device manufacture	Yes – Changes in weight bearing devices	Yes - Observation of experienced	Yes – Performance of clay and reed	Yes - Improvements occurred over	No	Diversification was possible but not formally analysed	No	No	No

	(from reed and clay)	design	participants by naïve participants	devices increased	multiple generations					
Kempe & Mesoudi 2014	Jigsaw puzzle solving	Yes – Additional pieces were correctly connected	Yes - Participants were provided with the output of previous participants	Yes – Number of pieces solved increased	Yes – Number of pieces solved increased over time.	No	No – Only one way to complete the whole puzzle	No	No	No
Muthukrishna et al. 2014	Image editing and knot tying tasks	Image editing task: Yes – Changes in tools/actions used to recreate an image. Knot tying task: No – Participants had to replicate a seeded skill	Yes - Participants were provided with the output of previous participants	Image editing task: Yes – Image editing skills increased. Knot tying task: No – Participants could only do worse than the initial demonstrator	Image editing task: Yes – Image editing skills increased over multiple periods. Knot tying task: No	No	No – In both experiments participants had to produce a single and pre-determined solution	Some evidence that participants were combining information from multiple models.	No	No
Derex & Boyd 2015	Virtual totem pole production	Yes – New tools were produced and new totem design were introduced	Yes – Mostly pay-off biased social learning	Yes – New tools and high score totems were produced	Yes – Totem designs were refined across time (and were associated with the production of new tools).	Yes - The production of complex tools was contingent on the discovering of simpler tools	Diversification was possible but not formally analysed	Yes – In partially isolated groups	Yes – Some tools had multiple functions	Yes – Discoveries opened up new discoveries not accessible at the start
Zwirner & Thornton 2015	Basket production from a predetermined list of materials	Yes – Changes in baskets design	Yes - Participants were provided with the output of previous participants	Yes - More efficient baskets were produced	Yes - Baskets efficacy increased over multiple generations	No	Diversification was possible but not formally analysed	No	No	No
Derex & Boyd 2016	Virtual remedy production	Yes – New remedies were produced	Yes – Mostly pay-off biased social learning	Yes – New active ingredients were produced and remedies scores got improved	Yes – Remedies scores increased over time.	Yes – The production of some active ingredients was contingent on the discovering of other active ingredients	Yes – Groups converged on different local optima	Yes – Individuals occasionally moved between partially isolated groups allowing recombination	No	Yes – Discoveries opened up new discoveries not accessible at the start
McGuigan et al. 2017	Puzzle box with multiple solutions	Yes – New tools were used	Yes – Mostly pay-off biased social learning	Yes – New and higher rewards were obtained	Yes – The number of higher rewards increased across time	Yes – Higher rewards were obtained by building upon previous	Some evidence of homogeneity of solutions within groups. Between-group variation was	No – Some individuals combined initial materials, but all these materials	No	No – All solutions could be produced from initial materials

						solutions	not formally analysed	were available at the beginning of the experiment		
Fay et al. 2018	Communication and reproduction of a route on a map	Yes – New descriptive elements were introduced	Yes - Participants were provided with the output of previous participants (and could directly interact in one treatment)	Yes - Reproduction accuracy increased	Yes - Cumulative refinements of the linguistic procedures used and transmitted and improvements in route reproduction accuracy across generations	No	No – Only one way to reproduce the correct route	No	No	No

References for Table S4

Beppu A, Griffiths TL. 2009 Iterated learning and the cultural ratchet. In Proceedings of the Annual Meeting of the Cognitive Science Society 31, 2089–2094.

Caldwell CA, Millen AE. 2008 Experimental models for testing hypotheses about cumulative cultural evolution. Evolution and Human Behavior 29, 165–171.

Derex M, Boyd R. 2015 The foundations of the human cultural niche. Nature Communications 6, 8398.

Derex M, Boyd R. 2016 Partial connectivity increases cultural accumulation within groups. Proceedings of the National Academy of Sciences 113, 2982–2987.

Derex M, Godelle B, Raymond M. 2013 Social learners require process information to outperform individual learners. Evolution 67, 688–697.

Fay N, Ellison TM, Tylén K, Fusaroli R, Walker B, Garrod S. 2018 Applying the cultural ratchet to a social artefact: The cumulative cultural evolution of a language game. Evolution and Human Behavior 39, 300-309.

Kempe M, Mesoudi A. 2014 An experimental demonstration of the effect of group size on cultural accumulation. Evolution and Human Behavior 35, 285–290.

Kirby S, Cornish H, Smith K. 2008 Cumulative cultural evolution in the laboratory: An experimental approach to the origins of structure in human language. Proceedings of the National Academy of Sciences 105, 10681–10686.

McGuigan N, Burdett E, Burgess V, Dean L, Lucas A, Vale G, Whiten A. 2017 Innovation and social transmission in experimental micro-societies. Philosophical Transactions of the Royal Society B 372, 20160425.

Mesoudi A. 2008 An experimental simulation of the 'copy-successful-individuals' cultural learning strategy: Adaptive landscapes, producer-scrounger dynamics and informational access costs. Evolution and Human Behavior 29, 350–363.

Muthukrishna M, Shulman BW, Vasilescu V, Henrich J. 2014 Sociality influences cultural complexity. Proceedings of the Royal Society B 281, 20132511.

Wasielewski H. 2014 Imitation is necessary for cumulative cultural evolution in an unfamiliar, opaque task. Human Nature 25, 161–179.

Wisdom TN, Goldstone RL. 2010 Social learning and cumulative mutual improvement in a networked group. In Proceedings of the Annual Meeting of the Cognitive Science Society 32.

Zwirner E, Thornton A. 2015 Cognitive requirements of cumulative culture. Scientific Reports 5, 16781.
